# Evidence that 2-hydroxyglutarate is not readily metabolized in colorectal carcinoma cells

**DOI:** 10.1186/s40170-015-0139-z

**Published:** 2015-12-01

**Authors:** Susan J. Gelman, Nathaniel G. Mahieu, Kevin Cho, Elizabeth M. Llufrio, Timothy A. Wencewicz, Gary J. Patti

**Affiliations:** Department of Chemistry, Washington University, St. Louis, MO 63130 USA; Department of Medicine, Washington University School of Medicine, St. Louis, MO 63110 USA

**Keywords:** 2-Hydroxyglutarate, Cancer metabolism, Mass spectrometry, Metabolomics, Isotope-based metabolomics, ^13^C-tracing

## Abstract

**Background:**

Two-hydroxyglutarate (2HG) is present at low concentrations in healthy mammalian cells as both an L and D enantiomer. Both the L and D enantiomers have been implicated in regulating cellular physiology by mechanisms that are only partially characterized. In multiple human cancers, the D enantiomer accumulates due to gain-of-function mutations in the enzyme isocitrate dehydrogenase (IDH) and has been hypothesized to drive malignancy through mechanisms that remain incompletely understood. While much attention has been dedicated to identifying the route of 2HG synthesis, the metabolic fate of 2HG has not been studied in detail. Yet the metabolism of 2HG may have important mechanistic consequences influencing cell function and cancer pathogenesis, such as modulating redox potential or producing unknown products with unique modes of action.

**Results:**

By applying our isotope-based metabolomic platform, we unbiasedly and comprehensively screened for products of L- and D-2HG in HCT116 colorectal carcinoma cells harboring a mutation in *IDH1*. After incubating HCT116 cells in uniformly ^13^C-labeled 2HG for 24 h, we used liquid chromatography/mass spectrometry to track the labeled carbons in small molecules. Strikingly, we did not identify any products of 2HG metabolism from the thousands of metabolomic features that we screened. Consistent with these results, we did not detect any significant changes in the labeling patterns of tricarboxylic acid cycle metabolites from wild type or IDH1 mutant cells cultured in ^13^C-labeled glucose upon the addition of L, D, or racemic mixtures of 2HG. A more sensitive, targeted analysis revealed trace levels of isotopic enrichment (<1 %) in some central carbon metabolites from ^13^C-labeled 2HG. However, we found that cells do not deplete 2HG from the media at levels above our detection limit over a 48 h time period.

**Conclusions:**

Taken together, we conclude that 2HG carbon is not readily transformed in the HCT116 cell line. These data indicate that the phenotypic alterations induced by 2HG are not a result of its metabolic products.

## Background

Two-hydroxyglutarate (2HG) is an endogenous metabolite that can occur naturally as either the L-(S)-enantiomer or the D-(R)-enantiomer [1]. Both enantiomers occur at low concentrations in healthy, mammalian cells and can be detected in patient biofluids [2]. The metabolic pathways responsible for producing low concentrations of 2HG in healthy cells remain incompletely characterized, but promiscuous utilization of alpha-ketoglutarate by phosphoglycerate dehydrogenase, lactate dehydrogenase A, and malate dehydrogenase has been implicated [3–5]. Production of low levels of 2HG in healthy cells has thus been hypothesized to be a result of unwanted side reactions, although recent evidence suggests that 2HG in healthy cells may have some regulatory functions [5–7].

It is thought that when small amounts of 2HG are produced in healthy cells, the 2HG is metabolized by L-2HG dehydrogenase or D-2HG dehydrogenase [8]. These 2HG dehydrogenases transform 2HG into alpha-ketoglutarate and have been referred to as a type of “house-cleaning enzyme.” Indeed, defects in either dehydrogenase lead to rare inborn errors of metabolism in which pathological levels of 2HG accumulate. More recently, the observation was made that D-2HG also accumulates in cancer cells which harbor gain-of-function mutations in isocitrate dehydrogenase (IDH) 1 and 2 [9]. It has now been shown that D-2HG producing *IDH1* and *IDH2* mutations occur in many glioma, acute myeloid leukemia, chondrosarcoma, cholangiocarcinoma, and T cell angioimmunoblastic lymphoma cancers [10]. Interestingly, D-2HG accumulates in these cancers despite the presence of a presumably functional D-2HG dehydrogenase.

Increased concentrations of L-2HG and D-2HG both lead to altered cellular phenotypes [8]. D-2HG is sometimes referred to as an “oncometabolite” because its accumulation is thought to contribute to cancer pathogenesis [11]. Most important to the current study, we note that the mechanisms by which L-2HG and D-2HG regulate cellular physiology are not well understood. While increased L-2HG and D-2HG both have been shown to inhibit alpha-ketoglutarate-dependent dioxygenase enzymes that modify chromatin, it has also been suggested that D-2HG drives cancer pathogenesis by other non-epigenetic mechanisms that have yet to be characterized [5, 12–16]. Additionally, the proposed epigenetic mechanisms alone do not adequately address the context dependency and dose specificity of accumulated 2HG on cellular physiology [11].

To better understand the mechanisms by which elevated 2HG influences cellular physiology, the objective of this work was to explore the possibility that 2HG is transformed into unexpected products. By applying cutting-edge metabolomic technologies, thousands of small molecules can be quantitatively assessed [17]. Some of these small molecules correspond to unknown metabolites that have not been characterized with respect to structure, pathway, and biochemical function [18, 19]. Given that products of 2HG may have metabolic effects themselves, mapping 2HG metabolism is an important advance in understanding 2HG biology. In addition to tracking 2HG carbon into potentially unexpected metabolites, we also sought to assess the transformation of 2HG into the tricarboxylic acid (TCA) cycle via alpha-ketoglutarate as we speculated that this reaction could regulate cellular phenotype by modulating redox potential. Notably, in the HCT116 colorectal carcinoma cells that we studied here, we did not find evidence of significant L-2HG or D-2HG metabolism over a 48 h time course.

## Methods

### Materials

All liquid chromatography solvents and additives were obtained from Sigma-Aldrich (St. Louis, MO), as well as all chemicals needed for the synthesis of 2HG. Stable isotope-labeled alpha-ketoglutarate was purchased from Cambridge Isotope Laboratories (Tewksbury, MA). Cell culture media and reagents were purchased from Life Technologies (Grand Island, NY), and IDH1 (R132H/+) cells were purchased from Horizon Discovery Ltd. (Cambridge, UK). An enzyme-linked immunosorbent assay (ELISA) kit for measuring D-2HG dehydrogenase was purchased from Cloud Clone Corp. (Houston, TX).

### 2HG synthesis

Racemic uniformly ^13^C-labeled 2HG (U-^13^C 2HG) was synthesized by reducing one equivalent of U-^13^C-labeled alpha-ketoglutarate with three equivalents of sodium borohydride in water. The alpha-ketoglutarate was first dissolved in water and cooled to 0 °C in an ice bath, then sodium borohydride was slowly added as a solid over approximately 10 min. The mixture was warmed to room temperature and stirred for 2 h, then quenched with an equal volume of 1 M HCl, and dried via rotary evaporation under reduced pressure. To remove excess borates, the resulting solid was suspended in high performance liquid chromatography (HPLC) grade methanol, filtered, concentrated to dryness, and stored at −20 °C. The structure and purity of synthetic U-^13^C 2HG was confirmed by ^1^H-NMR and LC/MS/MS. Trace amounts of fully labeled starting material were detected by LC/MS/MS. These were present at >1000-fold lower levels than U-^13^C 2HG and were too low in concentration to be detected by ^1^H-NMR.

### Cell culture

Wild type HCT116 and HCT116 R132H/+ cells were cultured in McCoy’s 5A Modified Medium with 10 % fetal bovine serum (FBS) and no antibiotics, at 37 °C with 5 % CO_2_. For uniformly ^13^C-labeled glucose (U-^13^C glucose) experiments, cells were grown in glucose-free DMEM supplemented with either 25 mM U-^13^C glucose or 25 mM non-labeled glucose. Cells were seeded in a 10-cm plate at a density of approximately 2 × 10^6^ cells in 10 mL of media.

### Protein assay

An ELISA experiment was performed to test for the presence of D-2HG dehydrogenase in HCT116 R132H/+ cells. HeLa cells, which have been reported to have D-2HG dehydrogenase, were used as a reference control [20]. Samples were prepared by centrifuging trypsinized cells, re-suspending in ice-cold phosphate buffered saline (PBS), and centrifuging again. The final cell pellet was re-suspended in 1 mL of ice-cold PBS and subjected to three repeated freeze-thaw cycles to break apart cellular components. After the final thaw, the sample was centrifuged and the supernatant was collected for analysis.

### Isotope tracing

HCT116 R132H/+ cells were plated 24 h prior to being administered U-^13^C 2HG. After 24 h, cells were grown in media supplemented with 10 % of 50 mM U-^13^C 2HG in PBS or 10 % of 50 mM non-labeled 2HG in PBS (yielding a final concentration of 5 mM 2HG) and harvested after a total of 48 h. When harvesting, medium was first aspirated then flash frozen in liquid nitrogen. Cells were washed with PBS three times, then HPLC grade water once, and quenched with 1 mL of ice-cold HPLC grade methanol. Cells were then scraped from the plate in methanol, pelleted, and dried via SpeedVac and lyophilization. The dry samples were weighed and normalized by dry mass. Samples were extracted by methods as we have described previously [21, 22]. Briefly, lyophilized cell pellets were re-suspended in methanol/acetonitrile/water (2:2:1) at 1 mL of solvent per 1 mg dry cell mass. After extraction, supernatants were dried on a SpeedVac and re-suspended in acetonitrile/water (1:1). The final solvent volume was normalized on the basis of dry mass, with a ratio of 100 μL per 1 mg dry cell mass.

### Measuring 2HG dilution of ^13^C-glucose labeling

HCT116 parent cells and HCT116 R132H/+ cells were plated 36 h prior to being administered exogenous non-labeled 2HG and U-^13^C glucose. After 36 h, cells were grown in media supplemented with 10 % FBS and 25 mM of either U-^13^C glucose or non-labeled glucose. At this time, cells were treated with either (i) 10 % of 25 mM L- or D-2HG, (ii) 10 % of 50 mM L- or D-2HG, or (iii) 10 % of 50 mM L- and D-2HG. Cells were then harvested after a labeling period of 12 h and extracted as described above.

### Liquid chromatography/mass spectrometry

Following extraction, 5 μL of each sample was injected onto a Luna Aminopropyl column (3 μm, 150 mm × 1.0 mm I.D., Phenomenex, Torrance, CA) connected to an Agilent 1260 capillary HPLC system (Santa Clara, CA) set to a flow rate of 50 μL/min. Mobile phase A consisted of 95 % water, 5 % acetonitrile (ACN), 20 mM ammonium hydroxide, and 20 mM ammonium acetate. Mobile phase B consisted of 95 % ACN and 5 % water. The column was kept at room temperature while the following linear gradients were applied: 0–5 min, 100 % B; 5–45 min, 100–0 % B; 45–50 min, 0 % B; 50–51 min, 0–100 % B; and 51–60 min, 100 % B. MS detection was performed by using an Agilent 6540 Q-TOF with an electrospray ionization (ESI) source and Agilent Jet Stream technology, mass range 50–1700 *m/z*. The following source parameters were used: drying gas temperature 300 °C, drying gas flow 9 L/min, nebulizer pressure 35 psi, sheath gas temperature 350 °C, sheath gas flow 11 L/min, capillary voltage 3000 V, and nozzle voltage 1000 V. A fragmentor voltage of 175 V was applied. Conventional liquid chromatography/mass spectrometry (LC/MS)-based metabolomic analyses were performed in negative ionization mode only. After introduction of labeled 2HG to cells, metabolites were extracted and profiled to identify possible 2HG products in both negative and positive ionization modes. Metabolite identifications were confirmed by comparing experimental retention time and fragmentation data to model standards. With the methods applied, citrate and isocitrate could not be resolved.

For improved sensitivity, we also performed targeted analysis of some key metabolites (alpha-ketoglutarate, fumarate, malate, and citrate) and evaluated isotopic enrichment manually. Targeted analyses were performed by injecting 3 μL of sample onto a Luna Aminopropyl column (same as described above) connected to a Dionex UltiMate 3000 UHPLC system, set to a flow rate of 50 μL/min with the same gradient as stated above. MS detection was performed with a Thermo Scientific Q Exactive Plus bench-top quadrupole-Orbitrap mass spectrometer in negative ionization mode at 140,000 resolving power by using selected ion monitoring (SIM).

Quantification of 2HG and glutamine was performed on an Agilent 6490 triple quadrupole instrument in negative ion mode, using multiple reaction monitoring (MRM). Sample aliquots of 0.3 μL were injected onto an Acquity UPLC BEH Amide column (1.7 μm, 2.1 × 50 mm I.D., Waters Corporation, Milford MA) connected to an Agilent 1290 Infinity UHPLC system set to a flow rate of 0.6 mL/min. Mobile phase A consisted of 50 % water, 50 % ACN, 0.4 % ammonium hydroxide, and 10 mM ammonium acetate. Mobile phase B consisted of 95 % ACN and 5 % water. The following linear gradient was applied: 0–1.0 min, 85 % B; 1.0–1.35 min, 60 % B; 1.35–1.4 min, 50 % B; 1.4–1.8 min, 50 % B; 1.8–3.0 min, 75 % B; and 3.0–6.0 min, 100 % B. The following source parameters were used: drying gas temperature 125 °C, drying gas flow 13 L/min, nebulizer pressure 55 psi, sheath gas temperature 400 °C, sheath gas flow 12 L/min, capillary voltage 2000 V, and nozzle voltage 0 V. For 2HG, the quantifier ion transition *m/z* 147.0 → 129.0 and the qualifier 147.0 → 101.0 were used with a fragmentor voltage of 100 V, a collision energy of 10 V, and a cell accelerator voltage of 3 V. For glutamine, the quantifier ion transition *m/z* 145.0 → 127.0 and the qualifier 145.0 → 109.0 were used with a fragmentor voltage of 120 V, a collision energy of 10 V, and a cell accelerator voltage of 3 V.

### Measuring 2HG consumption rate from media

HCT116 (parent) cells were plated in 3 mM unlabeled D-2HG and cultured for 48 h. LC/MS analysis was used to measure 2HG and glutamine in the media at 0, 24, and 48 h. The concentration of 2HG and glutamine was quantified at all time points using a standard curve to determine the rate of consumption. This was compared to plates containing only media (no cells) to account for non-cellular degradation occurring over the given time period. For this assay, we ignored intracellular contributions of 2HG and glutamine as they were negligible compared to the levels of 2HG and glutamine in the media. Media samples were extracted as described above.

### Data processing

Raw data was converted to mzXML by using msconvert and then analyzed with the XCMS software (peakwidth = 5–140 s and ppm = 12) [23]. For isotope analysis, our in-house X^13^CMS software package was utilized and isotopic labeling patterns were identified with the following parameters: mass of ^12^C = 12.000000 Da, mass difference between ^13^C and ^12^C = 1.003355 Da, ppm = 20, RTwindow = 10 s, and noiseCutoff = 8000 ion counts [24]. Some key metabolites (e.g., the TCA cycle intermediates) were also inspected manually.

### Statistical analysis

All experiments were performed in replicates of three (*n = 3* per group). Each labeling experiment was performed in parallel with pairs of equivalent non-labeled cultures. Samples were evaluated with a Student’s paired *t* test, with acceptable *p* values being less than 0.05.

## Results

### Global metabolomic comparison of IDH1 mutant cells to wild type controls

**Fig. 1:**
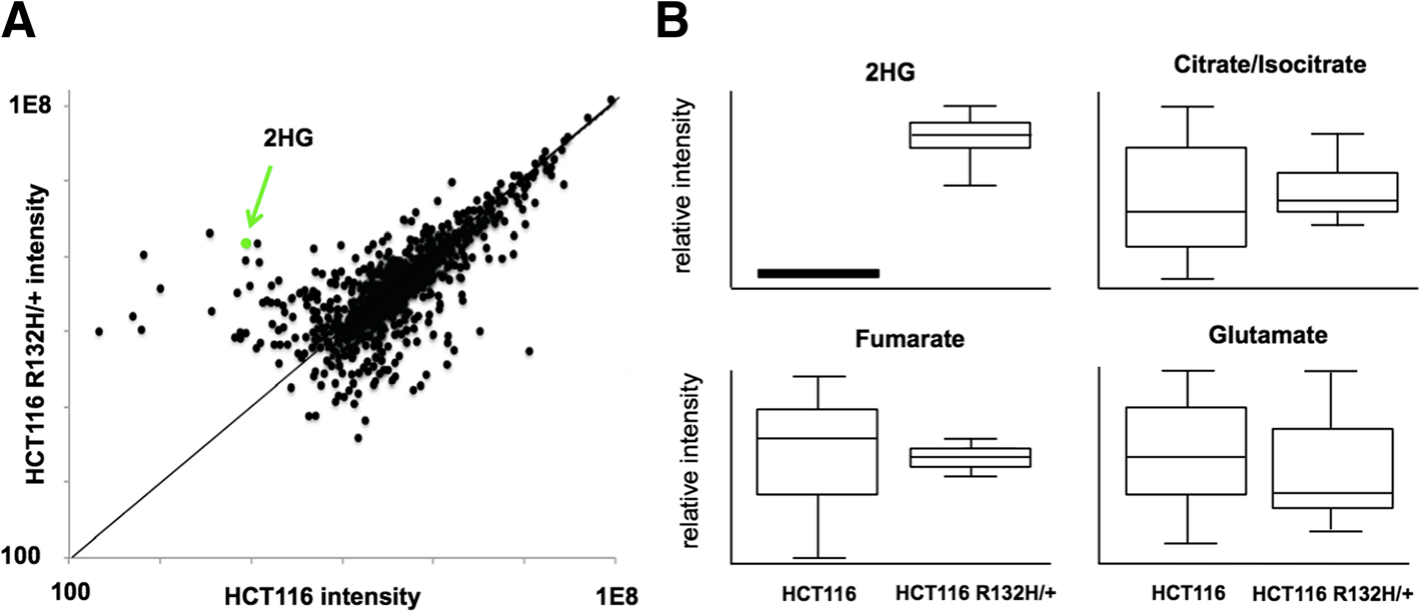
Conventional untargeted metabolomic comparison of parent HCT116 colorectal carcinoma cells to isogenic cells with heterozygous knock-in of the R132H IDH1 mutant. Results are based on *n = 3* samples per group. **a** Each circle displayed represents a metabolomic feature detected. Features off the y = x line have altered intensities between groups. Out of the 12,481 features detected, we found that 1722 were altered with *p* values <0.05 and fold changes >1.5. These data support that mutant IDH1, perhaps through the production of D-2HG, affects the metabolic phenotype of colorectal carcinoma cells and therefore are a reasonable choice to study 2HG metabolism. We identified the feature shown in green as 2HG, which was increased in HCT116 cells by a fold change of 165 to approximately 3.5 mM. **b** Box plots comparing the levels of selected metabolites between IDH1 mutant and wild type cells

This study focuses on one cell line, HCT116 colorectal carcinoma. To first assess the potential effects of 2HG on these particular cells, we compared the parent HCT116 cell line to an isogenic cell line with heterozygous knock-in of a mutant allele of *IDH1* (R132H). By using conventional LC/MS-based metabolomics, we detected 12,481 features from the cells with our in-house XCMS/Warpgroup software [25]. Here, we define a feature as an ion detected with a unique mass-to-charge ratio and retention time [17]. We found that 991 of the detected features had increased intensities and 731 had decreased intensities in IDH1 mutant cells relative to the parent cells, with a *p* value <0.05 and a fold change >1.5 (Fig. 1). As expected, a feature identified as 2HG was found to be increased with a fold change of 165 in the IDH1 mutant cells relative to the parent cells (see green arrow in Fig. 1). The results confirm that the *IDH1* mutation has a metabolic effect in these cells. We hypothesized that the altered features in IDH1 mutant cells were a result of the re-routing of nutrients for 2HG synthesis, products of 2HG metabolism, and/or effects of 2HG that are independent of its metabolism (e.g., enzymatic inhibition). The objective of this study was to find products of 2HG metabolism, which we predicted might be increased in the IDH1 mutant dataset from the current experiment. Given the complexity of metabolic regulation, however, it is not possible to identify 2HG products from these data directly. We note that no significant alterations were found in citrate/isocitrate, fumarate, and glutamate. These metabolites are expected to be closely upstream or downstream of 2HG based on pathway maps and therefore might have been predicted to be elevated if 2HG was being metabolized via this route. This result led us to further investigate which, if any, of the increased metabolic features in IDH1 mutant cells are products of 2HG transformation. To find products of 2HG transformation, we applied isotope-based metabolomic technologies as described below.

### Unbiased metabolomic screening identifies no products of 2HG metabolism

**Fig. 2:**
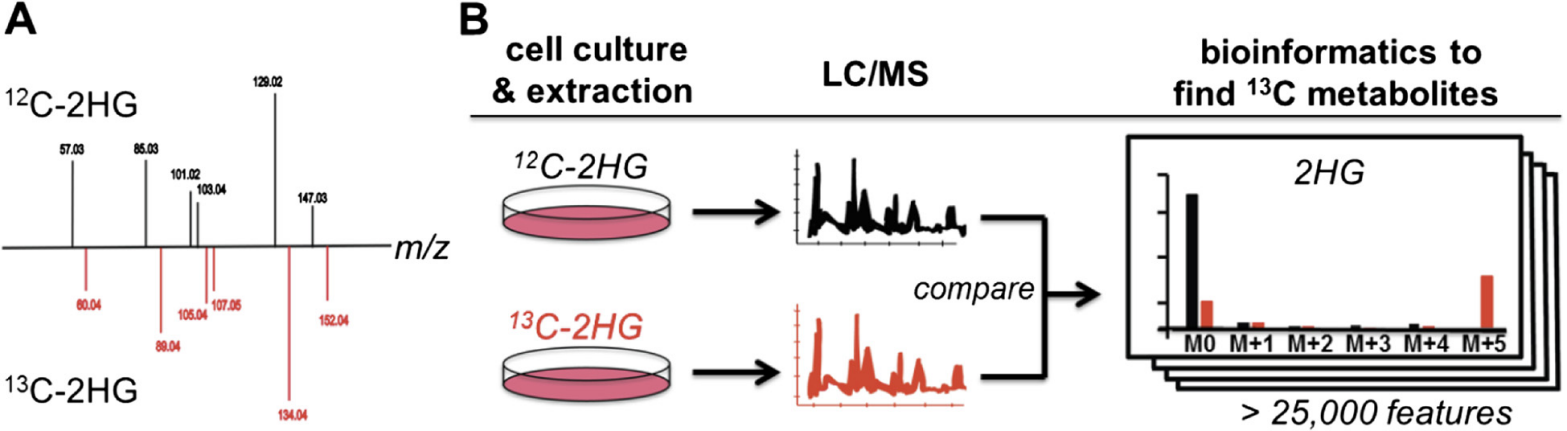
Strategy for tracking the fate of 2HG. **a** Successful synthesis of U-^13^C 2HG is supported by accurate mass measurements and tandem mass spectra. **b** Metabolomic platform for unbiased tracking of isotopes. Cells were grown in parallel cultures, with and without labeled substrate (*n* = 3 per group). Metabolites were extracted from the cells and analyzed by LC/MS. The data were processed by using our in-house X^13^CMS software, which is designed to unbiasedly find differences in isotopic enrichment. These differences correspond to labeled compounds that are possible biotransformations of the labeled substrate provided. Here, we show the isotopologue patterns for 2HG, which had an isotopic enrichment of 35 % in labeled samples. Metabolites of interest were identified by accurate mass, tandem mass spectra, and our isoMETLIN database

To track the fate of 2HG using our isotope-based metabolomic platform, we needed U-^13^C-enriched 2HG (Fig. 2). We synthesized U-^13^C 2HG from U-^13^C alpha-ketoglutarate obtained from Cambridge Isotope Laboratories as described in Methods. Successful synthesis of 2HG was validated by comparing the accurate mass, tandem mass spectra, and retention time of the synthesized compound to that of an authentic model compound purchased from Sigma-Aldrich (Fig. 2a). Our synthesis resulted in a racemic mixture of both U-^13^C L-2HG and U-^13^C D-2HG.

**Fig. 3:**
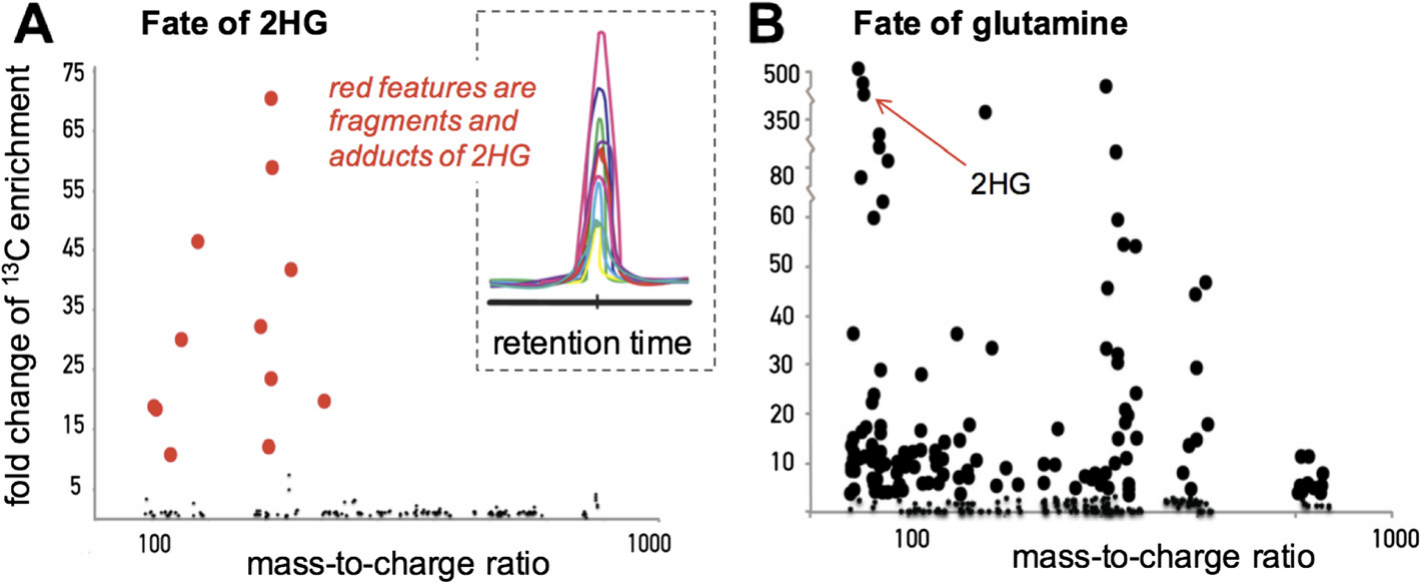
Global mapping of 2HG and glutamine fate. Plots showing altered metabolomic features in colorectal carcinoma cells after introduction of either **a** labeled U-^13^C 2HG (*n* = 3 per group) or **b** U-^13^C glutamine (*n* = 3 per group) for 24 h. The data shown are from colorectal carcinoma cells harboring mutations in *IDH1*. The y axis shows the fold change of isotopic enrichment calculated by dividing the intensity of each feature in the labeled samples by the intensity of each feature in the non-labeled samples. Features found to be enriched are shown with *enlarged circles* for clarity. Features containing isotopic label, shown in *red* on the left, were determined to be adducts or fragments of 2HG. The *inset* shows the extracted ion chromatogram (i.e., the LC trace) of each feature related to 2HG, each having the same retention time and chromatographic shape as expected for adducts and fragments. In contrast to the 2HG experiment where no labeled products were detected, the label from glutamine was incorporated into hundreds of features. One of the labeled features is annotated as 2HG. The glutamine data serve as a positive control that our platform can successfully identify biotransformations of a labeled substrate

**Fig. 4:**
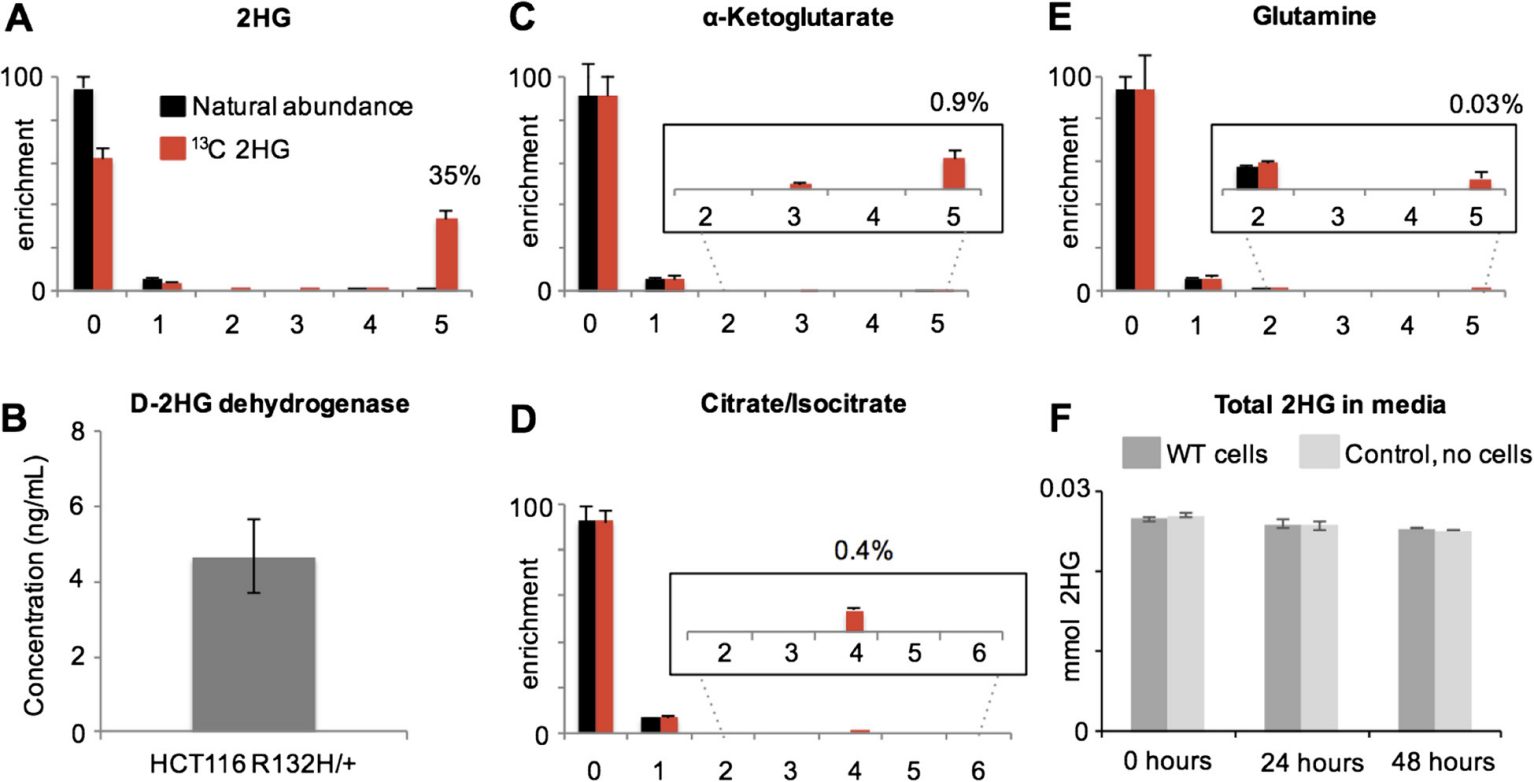
Exogenous 2HG enters cells, but is not metabolized at significant levels by 2HG dehydrogenase. **a** After treating HCT116 R132H/+ cells with U-^13^C 2HG for 24 h, we measured intracellular 2HG by mass spectrometry and found that 35 % was enriched. These data show that exogenous U-^13^C 2HG enters the cells. **b** An ELISA experiment confirmed that D-2HG dehydrogenase is present in HCT116 R132H/+ cells. **c**–**e** More sensitive targeted mass spectrometry methods were applied to measure low levels of isotopic enrichment in central carbon metabolites. **f** HCT116 parent cells were cultured in 3 mM D-2HG and compared to media preparations without cells as a control for spontaneous non-cellular degradation of 2HG. After 48 h, we found no statistically significant consumption of 2HG from the media (see Table 1). These data support that 2HG is not readily metabolized in HCT116 cells

Next, we gave HCT116 R132H/+ cells either U-^13^C 2HG or non-labeled 2HG, which was synthesized from non-labeled alpha-ketoglutarate by using the same preparative method (Fig. 3). Metabolites from cells treated with U-^13^C 2HG or non-labeled 2HG for 24 h were then isolated, analyzed by LC/MS, and the results compared by using the X^13^CMS software as described previously [24, 26, 27]. Multiplexed metabolite extractions were used to increase the number of compounds detected. Out of the more than 25,000 features screened, less than 20 were found to be different between the U-^13^C 2HG and non-labeled 2HG sample groups (Fig. 3a). One of the differences was found to be 2HG, which was enriched at the 35 % level (Fig. 4a). This result shows that 2HG was successfully transported into the cells. All other features found to be different between the sample groups had the same retention time and chromatographic profiles as 2HG, suggesting that they are in-source fragments/adducts of 2HG and not products of cellular 2HG metabolism [28]. This was verified by showing that a pure 2HG standard produced the same features when analyzed under identical LC/MS conditions.

These data suggest that neither L-2HG nor D-2HG are significantly metabolized over 24 h in colorectal cancer cells. As a positive control to demonstrate that our platform can effectively identify molecular transformations, we next fed the same colorectal carcinoma cells either U-^13^C glutamine or non-labeled glutamine for 24 h. We then isolated the metabolites, analyzed them by LC/MS, and used our X^13^CMS software to compare the labeled and non-labeled glutamine sample groups. In contrast to the 2HG experiments, we identified hundreds of metabolomic features that were altered between cells given labeled and non-labeled glutamine (Fig. 3b). The majority of these alterations had retention times unique from glutamine and corresponded to metabolites produced from glutamine carbon by cellular metabolism. As expected, one metabolite we identified as a product of glutamine was 2HG. Further analysis of glutamine transformation is beyond the scope of the current study, but the data can be viewed at the NIH metabolomics workbench or on our laboratory website (http://goo.gl/W4rbu1).

### Targeted screening of predicted 2HG products shows trace levels of enrichment

The enzyme D-2HG dehydrogenase converts D-2HG into alpha-ketoglutarate. Given that our unbiased metabolomic screen did not reveal any 2HG transformations, we wanted to validate that D-2HG dehydrogenase was present in our HCT116 R132H/+ cells. We confirmed the presence of D-2HG dehydrogenase by ELISA (Fig. 4b). The level of D-2HG dehydrogenase we measured in HCT116 R132H/+ cells was 4.7 ± 0.96 ng/mL, which was comparable to the value we measured in HeLa cells where D-2HG dehydrogenase has been previously reported [20].

To assess the possibility that D-2HG dehydrogenase converts labeled 2HG into labeled alpha-ketoglutarate at a concentration below the limit of detection of our global profiling platform, we performed a targeted LC/MS-based experiment and evaluated isotopic enrichment manually. Compared to global profiling analyses, targeted SIM experiments have improved sensitivity and therefore are able to better detect low levels of isotopic enrichment. We found that alpha-ketoglutarate was enriched in HCT116 R132H/+ cells given U-^13^C 2HG by approximately 1 % (Fig. 4c). We detected alpha-ketoglutarate with five ^13^C labels (0.9 %) and three ^13^C labels (0.1 %). The latter labeling pattern results from 2HG-derived alpha-ketoglutarate after one turn of the TCA cycle. We also detected trace amounts of labeling (<0.5 %) in other TCA cycle metabolites such as citrate/isocitrate and glutamine (Fig. 4d–e). We should point out that we cannot dismiss the possibility that some (or all) of this labeling results from U-^13^C alpha-ketoglutarate in our U-^13^C 2HG stock solution (i.e., residual starting material after synthesis) or spontaneous, non-cellular degradation of U-^13^C 2HG. Nonetheless, these labeling results place a ~1 % upper limit on the contribution of labeled 2HG to TCA cycle carbon at 24 h.

### Total accounting of net 2HG consumption from media

To evaluate the possibility that 2HG label is transformed into a metabolic product that we do not detect, we incubated parent HCT116 cells in 3 mM D-2HG for 48 h. After 24 and 48 h, we measured the total level of D-2HG remaining in the media. We did not detect a statistically significant decrease in the concentration of D-2HG between control media and media containing HCT116 cells at either time point (Fig. 4f). The small decrease in 2HG concentration between time points was not a result of cellular 2HG transformation but rather due to non-cellular 2HG degradation with time. In contrast, we observed a significant decrease in glutamine media levels over 48 h (Table 1). We determined that glutamine was consumed by cells at a rate of ~0.15 pmol/cell/h. We did not detect any statistically significant cellular consumption of 2HG above the detection limit of our assay (which we determined to be approximately 1 fmol/cell/h).

**Table 1:** 2HG and glutamine consumption in wild type HCT116 cells

	2HG	Glutamine
Consumption rate	Below detection of assay^a^	~0.15 pmol/cell/h
% Consumed	Below detection of assay	~97 %
*p* value	>0.2	<0.001

^a^We determined the detection limit of our assay to be approximately 1 fmol/cell/h

### Exogenous 2HG does not influence TCA cycle labeling patterns from U-^13^C glucose

**Fig. 5:**
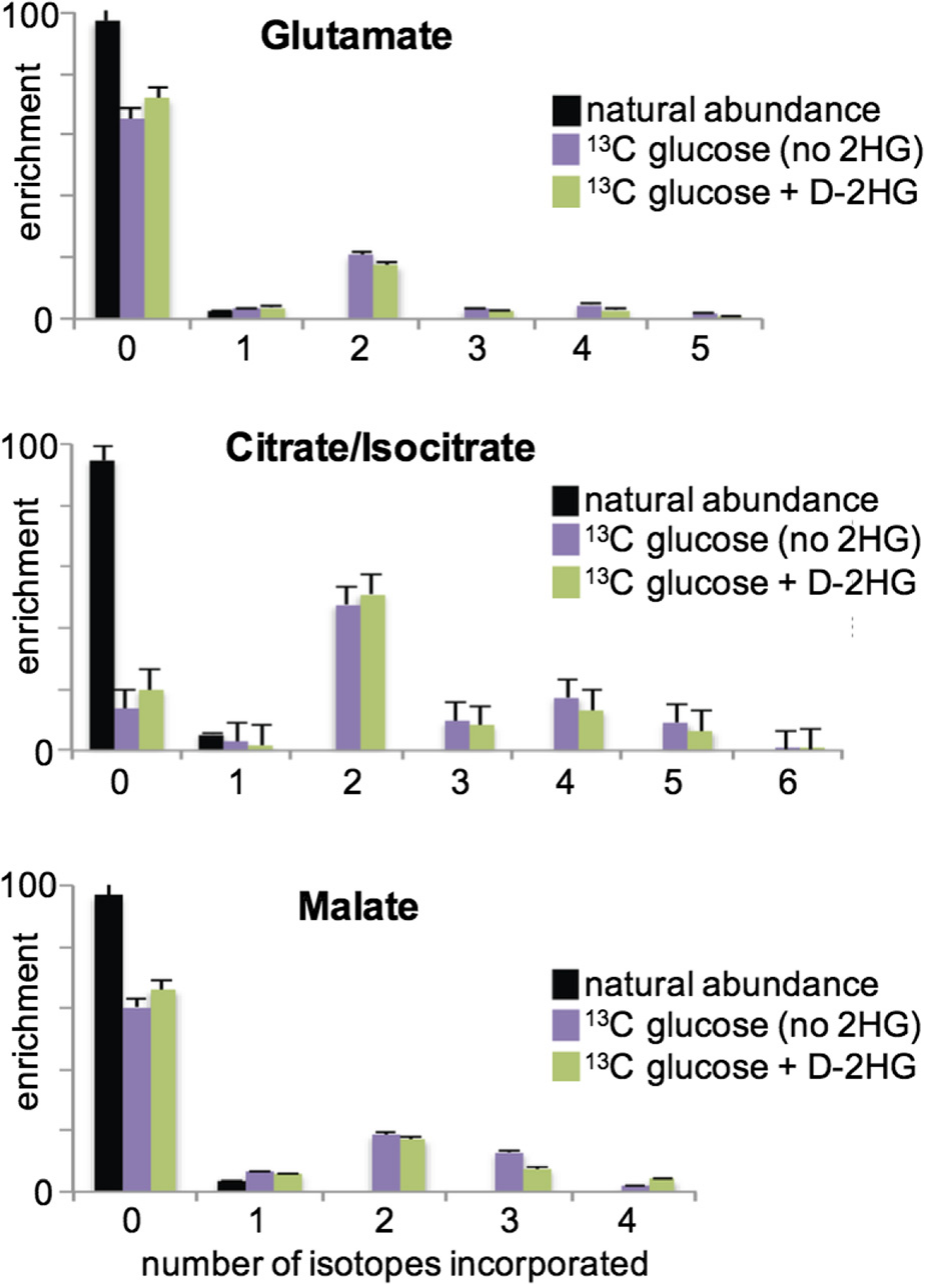
Exogenous 2HG does not influence TCA cycle labeling from U-^13^C glucose. As further support that significant amounts of 2HG are not metabolized into TCA cycle intermediates, we performed an experiment that had a reverse labeling scheme compared to that experiment shown in Fig. 2. U-^13^C glucose was given to cells and the labeling patterns of TCA cycle intermediates monitored as a function of L-2HG, D-2HG, or racemic mixtures of L- and D-2HG administration. Here, we show isotopologue distributions of metabolites from HCT116 R132H/+ cultures given U-^13^C glucose + D-2HG and from parent cultures given U-^13^C glucose and no 2HG. Isotopologue distributions were evaluated for TCA cycle intermediates and related compounds, three of which are displayed. No differences in isotopologue distributions were found to be statistically significant in these experiments (*n* = 3 per group) or those performed with the L-2HG enantiomer (*n* = 3 per group) and the racemic mixture of 2HG (*n* = 3 per group)

We predicted that U-^13^C 2HG given to colorectal carcinoma cells would be degraded by 2HG dehydrogenase enzymes to yield ^13^C-labeled alpha-ketoglutarate, which would then be metabolized by other pathways such as the TCA cycle. Yet, our metabolomic screens suggest that labels in U-^13^C 2HG are not significantly metabolized over a 24 h time course. To further support these results, we pursued a complementary U-^13^C glucose labeling experiment. In brief, we gave colorectal carcinoma cells U-^13^C glucose and then monitored labeling of TCA cycle intermediates when the cells were treated with 2HG for 24 h. We performed the experiments with either 2.5 or 5 mM of L-2HG, 2.5 or 5 mM of D-2HG, or a 5 mM racemic mixture of L-2HG and D-2HG. The labeling patterns of TCA cycle intermediates from U-^13^C glucose were not statistically different upon the addition of 2HG to the culture media (Fig. 5). Our results were comparable in the parent cells and cells harboring mutant IDH1. Since the isotopic labeling percentages of TCA cycle intermediates from U-^13^C glucose were not diluted by insertion of non-labeled 2HG carbons as alpha-ketoglutarate, the findings were consistent with the metabolomic data described above and support that 2HG is not significantly metabolized. Notably, TCA cycle intermediate labeling from U-^13^C glucose was not significantly different between cells that were given L-2HG, D-2HG, or racemic mixtures of both. This suggests that having a high concentration of both L-2HG and D-2HG simultaneously, such as the racemic mixture we synthesized, does not influence the carbon metabolism of either isomer.

It is important to clarify that the experiments described above involving U-^13^C glucose were not designed to assess the metabolic effects of 2HG. Although the isotopic enrichment plots in Fig. 5 show no significant differences in the labeling of central carbon metabolites upon treatment with 2HG for 24 h, these data do not provide information on absolute fluxes or relative pool sizes. It is not our intent to suggest that metabolism is unaffected by 2HG.

## Discussion

L-2HG and D-2HG occur naturally at low concentrations (<100 μM) in healthy mammalian cells [2]. In multiple cancers, the concentration of D-2HG increases substantially to mM levels as a result of gain-of-function mutations in *IDH* [10]. It has been shown that alterations in either L-2HG or D-2HG concentrations can have important functional consequences [7, 9]. Accordingly, much attention has been devoted to elucidating the biochemical routes of 2HG synthesis. However, the metabolic fate of 2HG has not been investigated comprehensively. Yet the pathway of 2HG transformation plays a relevant role in modulating 2HG levels and was therefore the focus of the current work. Additionally, we wanted to explore the possibility that 2HG is transformed into metabolic products with unique biochemical activities contributing to 2HG-related phenotypes.

We performed several separate experiments indicating that 2HG is not readily metabolized in colorectal cancer cells, independent of whether the cells harbor the R132H-IDH1 mutation. First, we fed cells U-^13^C 2HG and monitored thousands of metabolomic features for ^13^C label incorporation. After 24 h, we observed no labeled features other than those arising from 2HG (Fig. 3a). A more sensitive targeted analysis revealed trace levels of isotopic enrichment in some central carbon metabolites (Fig. 4c–e). By monitoring 2HG consumption, however, we showed that these transformations do not represent a statistically significant percentage of 2HG carbon (Fig. 4f). In an independent experiment, we fed cells U-^13^C glucose and monitored the isotopic labeling of TCA cycle intermediates upon addition of L-2HG, D-2HG, or racemic mixtures of both. We observed no significant labeling differences in cells treated with 2HG, indicating that any 2HG inserted into the TCA cycle is below the sensitivity limit of this experiment (Fig. 5).

The subject of how elevated 2HG levels contribute to cellular transformation and tumorigenesis has been an area of intense interest. The mechanism that perhaps has been best supported thus far is epigenetic regulation by inhibition of alpha-ketoglutarate-dependent enzymes. Given the structural and chemical similarity of 2HG to alpha-ketoglutarate, L-2HG and D-2HG can competitively inhibit alpha-ketoglutarate-dependent dioxygenases that modify chromatin [5]. These effects have been associated with alterations in growth and cellular differentiation [13]. This mechanism of 2HG-mediated epigenetic regulation is dependent upon the concentration of 2HG and has been well correlated with rates of 2HG synthesis [11, 15]. Our results are consistent with this model.

Another possible mechanism of 2HG action that has not been explored to date is that a downstream product of 2HG, possibly a previously uncharacterized metabolite, has unique biochemical activity. Our results do not support this model. By using untargeted metabolomic technologies, we screened over 25,000 features as potential 2HG products and obtained no leads. It may be possible that 2HG is metabolized to a compound not detected by LC/MS (e.g., macromolecules, low-concentration metabolites, etc.). However, we have shown previously that our metabolomic platform has broad coverage (for both polar and non-polar molecules) and high sensitivity for detecting metabolites with structures similar to 2HG as would be anticipated for the products of interest [21, 29]. Moreover, after 48 h, we have shown that there is no statistically significant consumption of 2HG above our assay detection limit of ~1 fmol/cell/h (Fig. 4e).

It is important to emphasize that our results are from only a single colorectal carcinoma cell line. Although we do not see evidence that 2HG is readily transformed here, such pathways may exist in other cell lines or cell types. Additionally, given that 2HG can be imported and exported from cells, it is also possible that 2HG produced from the tumors of whole animals is ultimately cleared from the organism even when the cancer cells producing 2HG cannot metabolize it.

## Conclusions

Intracellular concentrations of L-2HG and D-2HG are known to regulate cellular physiology by mechanisms that are incompletely understood. Critical to understanding how 2HG concentrations are controlled is the elucidation of pathways of 2HG synthesis and pathways of 2HG transformation. While the former has been investigated extensively, here we provide the first comprehensive assessment of the latter. Our results indicate that L-2HG and D-2HG are not readily metabolized in HCT116 colorectal carcinoma cells. Future work will explore the metabolism of 2HG in other cell types and culture conditions.
